# Qualitative Phytochemical Fingerprint and Network Pharmacology Investigation of *Achyranthes aspera* Linn. Extracts

**DOI:** 10.3390/molecules25081973

**Published:** 2020-04-23

**Authors:** Kouadio Ibrahime Sinan, Gokhan Zengin, Dimitrina Zheleva-Dimitrova, Ouattara Katinan Etienne, Mohamad Fawzi Mahomoodally, Abdelhakim Bouyahya, Devina Lobine, Annalisa Chiavaroli, Claudio Ferrante, Luigi Menghini, Lucia Recinella, Luigi Brunetti, Sheila Leone, Giustino Orlando

**Affiliations:** 1 Department of Biology, Science Faculty, Selcuk University, Campus, Konya, 42130 Konya, Turkey; sinankouadio@gmail.com (K.I.S.); gokhanzengin@selcuk.edu.tr (G.Z.); 2 Department of Pharmacognosy, Faculty of Pharmacy, Medical University-Sofia, 1431 Sofia, Bulgaria; dimizheleva@gmail.com; 3 Laboratoire de Botanique, UFR Biosciences, Université Félix Houphouët-Boigny, Abidjan 01, Cote d’Ivoire; 4 Institute of Research and Development, Duy Tan University, Da Nang 550000, Vietnam; mohamadfawzimahomoodally@duytan.edu.vn or; 5 Department of Health Sciences, Faculty of Science, University of Mauritius, Réduit 80837, Mauritius; devina.lobine@gmail.com; 6 Laboratory of Human Pathologies Biology, Department of Biology, Faculty of Sciences, and Genomic Center of Human Pathologies, Faculty of Medicine and Pharmacy, Mohammed V University in Rabat, Rabat 10106, Morocco; boyahyaa-90@hotmail.fr; 7 Department of Pharmacy, “G. d’Annunzio” University Chieti-Pescara, 66100 Chieti, Italy; annalisa.chiavaroli@unich.it (A.C.); luigi.menghini@unich.it (L.M.); lucia.recinella@unich.it (L.R.); luigi.brunetti@unich.it (L.B.); sheila.leone@unich.it (S.L.); giustino.orlando@unich.it (G.O.)

**Keywords:** *Achyranthes aspera*, antioxidant, fatty acids, enzyme inhibition, phytopharmaceutics, network pharmacology

## Abstract

*Achyranthes aspera* Linn. (Amaranthaceae), commonly known as the Prickly Chaff flower, is used as herbal medicine in the Ivorian’s culture, Africa. Nonetheless, there is currently a paucity of scientific information on *A. aspera* from the Ivory Coast. Herein, the antioxidant activity of *A. aspera* extracts (methanol, dichloromethane, ethyl acetate and infusion) as well as the enzymatic inhibitory potentials towards key enzymes in human diseases, namely Alzheimer’s disease, (cholinesterases: AchE and BChE), type 2 diabetes (α-glucosidase and α-amylase) and hyperpigmentation (tyrosinase) were assessed. The total phenolic (TPC) and flavonoid (TFC) content was determined using colorimetric methods and the individual compounds were characterized using ultra-high performance liquid chromatography coupled with hybrid quadrupole-Orbitrap high resolution mass spectrometry (UHPLC-HRMS). Furthermore, a network pharmacology analysis was conducted to predict putative targets of identified phenolic compounds. The highest TPC was observed in the infused extract (28.86 ± 0.12 mg GAE/g), while the dichloromethane extract (38.48 ± 1.48 mg RE/g) showed the highest level of TFC. UHPLC-HRMS analysis has revealed an abundance of fatty acids, flavonoids, phenols and acylquinic acids. Among tested extracts, the infused extract displayed the highest free radical quenching, reducing and metal-chelating ability. The extracts (except infusion) were effective as enzyme inhibitors against AChE, while only methanolic and infused extracts showed noteworthy anti-BChE effects. The methanolic extract showed a remarkable antityrosinase effect (56.24 ± 5.05 mg KAE/g), as well. Modest to moderate inhibitory activity was observed against α-amylase (all extracts) and α-glucosidase (only dichloromethane extract). Finally, the network pharmacology analysis suggested the carbonic anhydrase II enzyme as a putative target for explaining, at least in part, the traditional use of *A. aspera* preparations as diuretic and blood clotting agent. Data amassed herein tend to validate the use of *A. aspera* in traditional medicine, as well as act as a stepping stone for further studies in the quest for novel phytopharmaceuticals. In this context, it is desirable that this study will contribute to the validation of the traditional uses of this plant in the African herbal medicine, and to the valorization of the whole chain production of *A. aspera*, as a local and sustainable botanical resource.

## 1. Introduction

The burden of non-communicable diseases (NCDs) is rising swiftly in low-resourced countries, resulting in ill health, worsened poverty and poor social development. In Sub-Saharan Africa, NCDs are the second most common cause of mortality, accounting for 2.6 million deaths annually, which is equivalent to approximately 35% of all deaths in the region Yuyun, et al. [1]. Healthcare systems in the majority of Sub-Saharan Africa countries are fragile, fragmented, under-resourced, inaccessible and inefficient for a quick and effective response to rising burden of NCDs and hence managing these chronic diseases in Africa represents a huge challenge [2,3]. Thus, in line with the World Health Organization’s strategy, healthcare authorities in many low-resourced countries have been promoting a form of healthcare system that combines both traditional practices, predominantly the herbal traditional medicine, and conventional medicine to alleviate diseases. As many African countries, traditional medicine is deeply rooted in the Ivorian culture, and has remained as the primary healthcare system. Among different medicinal plants, *Achyranthes aspera* L., is reputed for its use in the folkloric medicine of Ivory Coast [4].

*A. aspera* (family Amaranthaceae), commonly recognized as Prickly Chaff flower (English), is an indigenous medicinal plant in Asia, South America and Africa. It is an erect annual herb that grows up to 0.3–1 m in height with stiff branches and thick and elliptic leaves. The plant bears greenish white flowers [5,6,7]. The plant is commonly harvested and used by traditional healers for alleviating fever, especially malarial fever, wound, blood clotting, dysentery and hypertension among others [5,8,9]. In the Ivory Coast, decoction preparations are also used to treat diabetes and as diuretic [4]. The plant is also used to cure tonsillitis, head wounds, ringworm, diarrhea and asthma in the Eastern Africa region [6].

Biological investigations have also revealed that *A. aspera* possess antibacterial [6,10], antifungal, thyroid-stimulating, antiperoxidative [7], anti-inflammatory [9,11], antiarthritic [11], immunomodulatory [12,13], wound healing [14], antiobesity [15], anticonvulsant [16], anticancer [8] and hepatoprotective [17] properties. Several classes of phytochemicals such as saponins, phenolic compounds, flavonoids, alkaloids, steroids and terpenoids [6,8,9,14,16] have been reported to occur in this plant. 

As far as our literature survey has showed, most of previous investigations have focused on *A. aspera* that grows in India, whereas there is still a dearth of scientific validation on the *A. aspera* that grows in the continent of Africa, which was investigated only for elucidating antimicrobial, anthelmintic and wound healing properties [18]. In this regard, the aims of the present study was designed to evaluate antioxidant capacity and inhibitory potential against key enzymes targeted in the management of Alzheimer’s disease, type 2 diabetes and skin hyperpigmentation disorders, of *A. aspera* that grows in the Ivory Coast. In addition, the phytochemical profile was determined using UHPLC-HRMS. Finally, a network pharmacology analysis was also carried out to predict putative targets of identified secondary metabolites, namely phenolic and flavonoid compounds. It is expected that this study will contribute to the validation of the traditional uses of this plant in the African herbal medicine, thus improving the local product chain, also in terms of sustainability.

## 2. Results and Discussion

### 2.1. Total Phenolic and Flavonoid Contents

**Figure 1:**
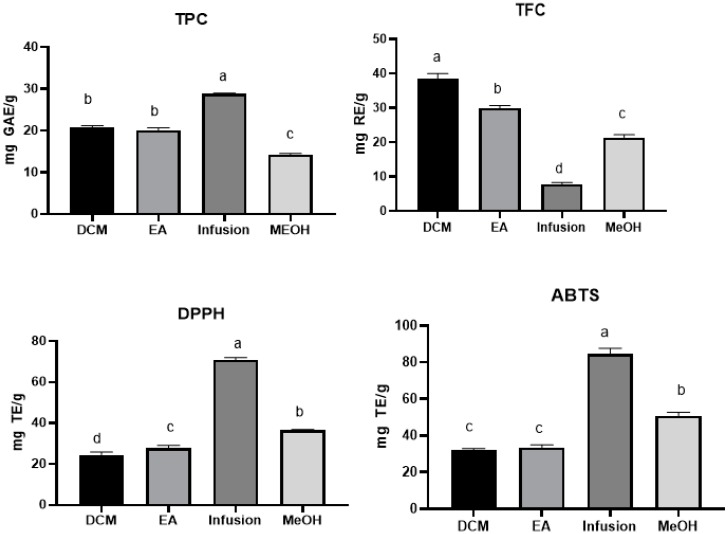
Total phenolic (TPC), flavonoid (TFC) content and radical scavenging abilities (1,1-diphenyl-2-picrylhydrazyl (DPPH·) radical and 2,2-azino-bis (3-ethylbenzothiazloine-6-sulfonic acid) radical cation (ABTS+) of the tested extracts. Values expressed are means ± S.D. of three parallel measurements. GAE: Gallic acid equivalent; RE: Rutin equivalent. TE: Trolox equivalent. Based on Tukey’s assay at *p* < 0.05, different letters (a, b, c and d) with in each bar indicated significant differences among the tested extracts (a: the highest value).

Plants are considered as a repository of molecules with biological properties that are useful for the modern drug discovery program. Among the known classes of bioactive compounds, polyphenols are well acknowledged for their potential as therapeutics [19,20,21]. Therefore, the present study evaluated the total phenolic content (TPC) and total flavonoid content of *A. aspera* extracts using spectrophotometric methods. Experimental data expressed as equivalents of gallic acid (GAEs), for TPC, and rutin (REs), for TFC, are summarized in Figure 1. The TPC varied from 14.28 ± 0.24 to 28.86 ± 0.12 mg GAE/g, with the highest content observed in the infused extract. The dichloromethane extract (38.48 ± 1.48 mg RE/g) followed by ethyl acetate (29.90 ± 0.71 mg RE/g) extract displayed the richest total flavonoid content. 

### 2.2. Characterisation of Metabolites in A. aspera Extracts

Based on retention times, MS and MS/MS accurate masses, fragmentation patterns and comparison with reference standards and literature data, a total of 54 metabolites were identified or tentatively annotated in *A. aspera* extracts (Table 1). 

Full details for compounds identified are given in Supplementary Materials. 

### 2.3. Fatty Acids

Four saturated, seven monounsaturated and twenty-one polyunsaturated fatty acids were tentatively identified in *A. aspera* extracts.

### 2.4. Saturated Free Fatty Acids

Compound **1** yielded a deprotonated ion at *m*/*z* 187.096 (C_9_H_16_O_4_) together with the fragment ions at *m*/*z* 169.086 ([M − H − H_2_O]^−^ and *m/z* 125.095 ([M − H − H_2_O − CO_2_]^−^ suggesting carboxylic groups. This fragmentation pathway was previously described [22] and 1 was identified as azelaic acid. In the same manner 2, 4 and 24 were tentatively identified as undecanedioic, dodecanoic and 9,10-dihydroxy-octadecanoic acids, respectively (Table 1).

### 2.5. Monounsaturated Free Fatty Acids

Concerning 3, the base peak at *m*/*z* 183.138 ([M − H − CO_2_]^−^, supported by the diagnostic ion at *m/z* 165.127 ([M − H − H_2_O − CO_2_]^−^ was indicative for the dodecenedioic acid (traumatic acid). Regarding 7, fragment ions at *m*/*z* 195.138 (C_12_H_19_O_2_) and 179.143 (C_12_H_19_O) demonstrated the position of the OH group at C-12, and double bond between C-2 and C-11. Due to the prominent ion at *m*/*z* 109.063 (C_7_H_9_O), 7 was tentatively assigned to 12-hydroxy-6-heptadecenoic acid. At the same manner based on prominent ion at *m*/*z* 155.063 (C_9_H_15_O_2_), 12 could be related to 9-hydroxyoctadecenoic acid. Isobaric pairs 22/23 and 31/32 shared the same [M − H]^−^ at *m*/*z* 313.238 and 329.233, respectively. 22 gave diagnostic ion at *m*/*z* 183.1382 ([C_11_H_19_O_2_]^−^) and 129.091 ([C_7_H_13_O_2_]^−^) indicating OH at C-12. Moreover, the ion at *m/z* 99.080 ([C_6_H_11_O]^−^) corresponded to another OH group at C-13. Thus, 22 was identified as 12,13-dihydroxy-9-octadecenoic acid [23]. Fragmentation pathway of 23 included ions at *m*/*z* 295.227 ([M − H − H_2_O]^−^) and 277.217 ([M − H − 2H_2_O]^−^) showed the presence of two hydroxyl groups. The fragment ion at *m*/*z* 201.11325 ([C_10_H_17_O_4_]^−^) formed from the neutral loss of octane group (C_8_H_16_) suggested that one hydroxyl group was linked to C-10, and the position of vinyl bond could be between C-11 and C-12 or between C-12 and C-13. Based on the fragment ion at *m*/*z* 171.103, ([C_9_H_15_O_3_]^−^) due to another hydroxyl group located at C-9, 23 was identified as 9,10-dihydroxy-12-octadecenoic acid [23]. 31 gave fragment ions at *m*/*z* 229.144 ([M − H − C_6_H_12_O])^−^, 211.133 ([M − H − C_6_H_12_O − H_2_O])^−^, 193.122 ([M − H − C_6_H_12_O − 2H_2_O])^−^ and 99.080 ([C_6_H_11_O]^−^) indicated three hydroxyl groups. The diagnostic ions at *m*/*z* 171.101 ([C_9_H_15_O_3_]^−^) and 139.111 ([M − H − C_9_H_15_O_3_ − H_2_O]^−^) further indicated that hydroxyl groups were located at C-9, C-10 and C-13. Thus, 31 was identified as 9, 10, 13-trihydroxy-11-octadenoic acid [23]. Similar fragmentation was observed in the MS/MS spectrum of 32 except the lack of fragments at *m*/*z* 229.144, and 193.122. The difference could be related to isomerization of OH group and double bond from C-10 to C-12 and 32 was tentatively assigned to 9,12,13-trihydroxy-10-octadecenoic acid [23] (Table 1).

### 2.6. Polyunsaturated Free Fatty Acids

Concerning 5 with [M − H]^−^ at *m*/*z* 277.217 (C_18_H_30_O_2_), the diagnostic ions at *m*/*z* 233.227 [M − H − CO_2_]^+^ allowed for the tentatively annotation of linolenic acid. The fragmentation pathway of 6 (C_17_H_28_O_3_) including fragment ions at *m*/*z* 157.086 (C_8_H_13_O_3_), 121.100 (C_9_H_13_) and 113.097 (C_7_H_13_O), could related the compound to 8-hydroxy-9,11,13-heptadecatrienoic acid. Isobaric pair 9/10 demonstrated similar fragmentation behavior. Based on the exact mass, molecular formula (C_18_H_30_O_3_) and the characteristic fragmentation patterns of hydroxypolyunsaturated fatty acids, 9/10 were identified as 13-hydroxy-9,11,15-octadecatrienoic acid and 15-hydroxy-9,11,13-octadecatrienoic acid, respectively. Compound 14 gave a deprotonated molecule [M − H]^−^ at *m*/*z* 307.191. The base peak at *m*/*z* 235.133 (C_14_H_19_O_3_) revealed a hydroxyl group at C-14. Abundant fragment ions at *m/z* 211.133 (C_12_H_19_O_3_; 34.15%), *m*/*z* 185.117 (C_10_H_17_O_3_; 84.61%) and *m*/*z* 121.064 (C_8_H_9_O; 87.72%) correlated to the position of three double bonds at C-11, C-13 and C-15 and a keto group at C-9. Accordingly, 14 were identified as 14-hydroxy-9-oxo-11,13,15-octadecatrienoic acid. Four isobars 15-18 shared the same [M − H]^−^ at *m*/*z* 309.207. Based on the different fragmentation patterns, compounds were identified as dihydroxyoctadecatrienoic acids. At the same manner, 19–21, 25–30 and 31–32 were related to dihydroxyoctadecadienoic and trihydroxyoctadecadienoic acids (Table 1).

### 2.7. Carboxylic, Phenolic and Acylquinic Acids, Phenylethanoid Glycosides and Flavonoids

One carboxylic (quinic acid, 37), five phenolic acids including three hydroxybenzoic acids (salicylic 33, protocatechuic 34 and gentisic acid 35), three hydroxycinnamic acids including (caffeic 36 and ferulic 38 acids), two monoacylquinic (chlorogenic 41 and 4-caffeoylquinic acid 42), two diacylquinic acids (3,5-dicaffeoylquinic 43 and 4,5-dicaffeoylquinic acids 44) and one triacylquinic acid (45) were found in the studied *A. aspera* extracts (Table 2). Moreover, two hexosides of salicylic (39) and gentisic acids (40) as well as six flavonoids (46–51) were identified. Most of compounds were identified by comparison with standard references and literature data. The acylquinic acids elucidation was based on the structure-diagnostic hierarchical keys for the identification of chlorogenic acids [26,27], while flavonoid dereplication was supported by the RDA cleavages of the flavonoid skeleton [28]. 

### 2.8. Other Compounds

Compound 52 [M − H]^−^ at *m*/*z* 385.188 gave fragment ions at *m/z* 223.123 [M − H − 165.05]^−^ and 205.12 [M − H − 165.05 − 18]^−^, corresponding to the subsequent losses of one hexose and hydroxyl group. On the other hand, diagnostic ions at *m*/*z* 153.090 (C_9_H_13_O_2_) and 71.012 (C_4_H_7_O) could be related to 4-hydroxy-3,5,5-trimethylcyclohex-2-enone and but-3-en-2-ol, respectively. Thus, 52 was assigned to isobaric pair roseoside/corchoionoside. Regarding 53, with [M − H]^−^ at *m*/*z* 333.168 (C_19_H_26_O_5_), consequent losses of H_2_O and CO_2_ at *m*/*z* 315.157 and 289.179 corresponded to the steroid structure. H_2_O molecule originates from the OH group at position 14, because the 2,3-diol functionality, the alternative source of H_2_O molecules, should be resistant to loss of H_2_O due to bridging of the proton between the vicinal diol oxygen [25]. This fact was unambiguously proved by the sustainability of the ortho-dihydroxyl group in other fragment ions. Two keto groups are deduced from the subsequent losses of CO_2_ ([M − H − 44]^−^) and HCHO ([M − H − 44 − 42]^−^) at *m/z* 247.170. Fragment ions at *m*/*z* 219.1140 (C_14_H_19_O_2_) and 135.043 (C_14_H_19_O_2_) corresponded to the cleavage of ring D and C, respectively while ring A was witnessed by *m*/*z* 122.0359 (C_7_H_6_O_2_). Accordingly, 53 could be associated with rubrosterone, recently isolated from *A. rubrofusca* [29] (Table 1). Compound 54 gave diagnostic fragment ions at *m*/*z* 461.292 ([M − H − H_2_O]^−^) and 403.249 ([M − H − C_3_H_8_O_2_]^−^), due to the loss of the OH group at C-25 and cleavage between C-23 and C-24 [25]. The prominent ion at *m*/*z* 319.192 suggested the loss of side chain from the steroid nucleus. The fragment ions at *m*/*z* 301.181 were due to the loss of the OH group at C-14, while the abundant ion at *m*/*z* 159.106 (41.82%) could be related to the side chain residue at C-17. In line with aforementioned, 54 was tentatively ascribed to 20-hydroxyecdysterone (Table 1).

The full MS fingerprint showed that the studied *A. aspera* extracts were particularly rich in fatty acids, phenolic compounds and ecdysteroids (Table 1). A total of 51, 47, 53 and 44 secondary metabolites were characterized in the ethyl acetate, methanolic, dichloromethane and infused extracts, respectively. Among them, 38 were found in all extracts. 

Thirty-two fatty acids, including 4 saturated, 7 monounsaturated and 21 polyunsaturated fatty acids were tentatively identified in *A. aspera*. Twenty two of them were presented in all extracts. Azelaic acid (1), a saturated C9-dicarboxylic acid was detected in all studied extracts except dichloromethane, while undecanedioic acid (2), 9,10-dihydroxy-11,13,16-octadecatrienoic acid (16) and 9,10-dihydroxy-octadecanoic acid (24) were identified only in organic extracts. Four fatty acids (6, 7, 8 and 12) were found only in ethyl acetate and dichloromethane extracts. 19 was detected in ethyl acetate, dichloromethane and infused extracts. Linolenic acid (5), an essential fatty acid with important physiological functions, was detected only in the ethyl acetate extract. Chakrabarti and colleagues [30] described the linolenic acid as a predominant constituent, in the petroleum ether extract of *A. aspera* seed.

Based on UHPLC-HRMS, one carboxylic acid, five phenolic acids, five acylquinic acids and two phenylethanoid glycosides were identified in *A. aspera*. Salicylic acid (33), caffeic acid (36), quinic acid (37) and ferulic acid (38) were found in all studied extracts. Chlorogenic acid (41), 3,5-dicaffeoylquinic acid (43) and 4,5-dicaffeoylquinic acid (44) were detected in all samples, while 4-caffeoylquinic acid (42) was observed only in dichloromethane and infused extracts. On the other hand, protocatechuic acid (34) and 3,4,5-tricaffeoylquinic acid (45) were detected only in the organic samples. Furthermore, the presence of gentisic acid (35) was identified in all extracts except the ethyl acetate, whereas its derivative, gentisic acid-*O*-hexoside (40), was detected only in methanolic and dichloromethane extracts.

Six flavonoids (49–51), glycosides of kaempferol, quercetin and apigenin were identified in most of studied extracts. Only tiliroside (51) was not found in the dichloromethane extract.

In addition, two ecdysteroids (53 and 54) were tentatively identified in all studied *A. aspera* extracts. Our finding is consistent with previous studies on *Achyrantes* species [31].

### 2.9. Antioxidant and Enzyme Inhibitory Effects

Samples of *A. aspera* deriving from four solvents with different polarity were characterized in terms of their biological activities, i.e., in-vitro antioxidant and enzyme inhibitory effects.

Oxidative stress occurs when there is a disturbance in the dynamic balance between reactive oxygen or nitrogen species (ROS/RNS) generation and endogenous antioxidant system, which leads to the massive accumulation of free radicals. This brings impairment to cells and tissues, for instance, by triggering structure modifications of DNA, lipids and proteins. Such impairments are known to lead to all kinds of diseases. In order to neutralize the excess of free radicals and to contribute to disease prevention, the intake of exogenous antioxidant is highly recommended [32,33]. Thus, in the past decades, researchers have highly focused in investigating plants to find effective and safer antioxidant compounds.

**Figure 2:**
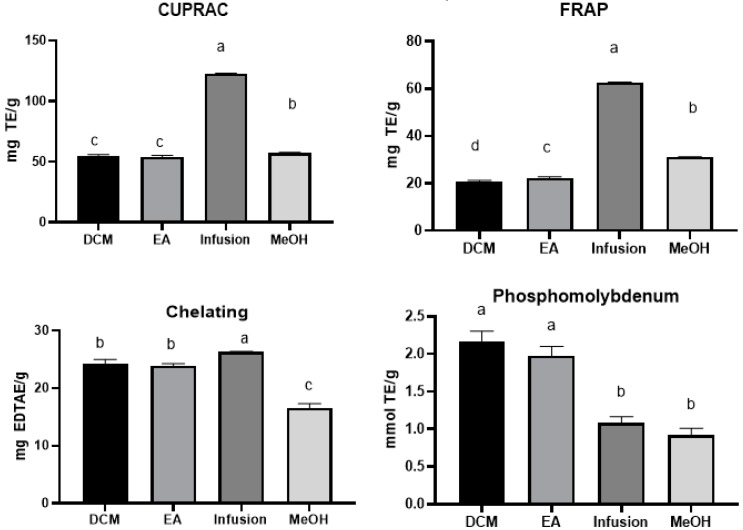
Reducing power (CUPRAC and FRAP), metal-chelating and phosphomolybdenum activities of the tested extracts. Values expressed are means ± S.D. of three parallel measurements. TE: Trolox equivalent; EDTAE: EDTA equivalent. Based on Tukey’s assay at *p* < 0.05, different letters (a, b, c and d) with in each bar indicated significant differences among the tested extracts (a: the highest value).

The antioxidant activity of the different *A. aspera* extracts was evaluated by in-vitro cell-free assays: free radical scavenging (on 1,1-diphenyl-2-picrylhydrazyl (DPPH·) radical and 2,2-azino-bis (3-ethylbenzothiazloine-6-sulfonic acid) radical cation (ABTS+)), reducing power (cupric ion reducing (CUPRAC) and ferric reducing antioxidant power (FRAP)) and total antioxidant (by phosphomolybdenum), and ferrous ion-chelating. The results are illustrated in Figure 2. Based on the experimental findings, it was observed that the infused and methanolic extracts have the highest free radical scavenging capacity and reducing power. For DPPH and ABTS assays, the activities varied from 24.45 ± 1.36 to 70.75 ± 1.22 mg TE/g and 32.20 ± 0.71 to 84.48 ± 3.24 mg TE/g, respectively. Regarding the reducing ability, the infused extract was the most potent with the mean values of 122.42 ± 0.52 and 62.59 ± 0.20 mg TE/g for CUPRAC and FRAP assays, respectively, with approximately two-fold higher than the values of the methanolic extract. 

Modest total antioxidant capacity was displayed by all extracts, whereby dichloromethane and ethyl acetate extracts (2.18 ± 0.13 mmol TE/g and 1.98 ± 0.12 mmol TE/g) exerted the highest activity. In terms of the ability to chelate metals, the highest activity was observed in the infused extract with a mean value of 26.31 ± 0.12 mg EDTAE/g. Overall, the infused extract was a superior source of antioxidant compounds, which is consistent with its total phenolic content.

The antioxidant activity of *A. aspera* extracts may be related to the contribution of more antioxidant bioactive compounds. In fact, the antioxidant activities of some compounds identified in *A. aspera* extracts were reported in several studies. As example, some compounds such as protocatechuic acid, caffeic acid, chlorogenic acid, ferulic acid and gentisic acid are referred to as a powerful antioxidant [34,35,36,37]. Previously, Yeh and Yen [38] reported the significant induction of hepatic antioxidant enzyme by gentisic acid, caffeic acid and ferulic acid, following oral administration of these compounds to male Sprague-Dawley rats. Additionally, the antioxidant effects of caffeoylquinic acid derivatives were reported [39]. According to Balasundram et al. [40], the antioxidant ability of phenolic compounds is hinged on the number and position of hydroxyl and aromatic groups in their structure. Based upon this information, we postulated that the antioxidant activities of *A. aspera* extracts might derive from phenolic acid compounds rather than other identified compounds.

*A. aspera*, due to its earlier related antioxidant constituents, may play a role in preventing the development and progression of certain human diseases including Alzheimer’s disease, type 2 diabetes mellitus and skin diseases, which are characterized by upregulation of multiple oxidative stress and inflammatory pathways. In this regard, enzyme inhibition represents a cornerstone in pharmacotherapy, and this is due to the mechanism of action of many drugs that, acting as inhibitors, are able to correct, albeit partially, the supraphysiological metabolic imbalances occurring in chronic diseases, especially those characterized by increased burden of inflammation and oxidative stress [41,42]. For example, the inhibition of cholinesterases (AChE and BChE) plays a pivotal role in the management of Alzheimer’s disease and this inhibition might increase the level of acetylcholine in the synaptic gap, which helps to improve cognitive functions of these patients [42]. In addition, the inhibition of α-amylase and α-glucosidase could improve blood glucose level control, in type 2 diabetes mellitus patients, after carbohydrate-rich meal intake [43]. Regarding the tyrosinase, it is a key enzyme involved in the synthesis of melanin and its inhibition could be crucial in alleviating hyperpigmentation related to inflammatory and infectious skin diseases [44].

Therefore, we assessed the enzyme inhibitory potential of *A. aspera* extracts against the aforementioned enzymes. The results are depicted in Table 2. All extracts, except the infused extract, were effective against AChE, with a value ranging from 3.28 ± 0.21 and 4.87 ± 0.10 mg GALAE/g (IC_50_: 0.55–0.82 mg/mL), while only infused (15.36 ± 0.22 mg GALAE/g; IC_50_: 0.55 mg/mL) and methanolic (16.00 ± 0.48 mg GALAE/g; IC_50_: 0.53 mg/mL) extracts showed remarkable anti-BChE effects. Despite there being an high TPC, the absence of protocatechuic acid or 3,4,5-tricaffeoylquinic acid in the infused extract might account for its inability to inhibit AChE. Protocatechuic acid is reported to possess antioxidant and neuroprotective effects, closely intertwined [45]. In fact, Adefegha and colleagues [34] showed that protocatechuic acid, by regulating cholinesterases and brain antioxidant enzyme activities, suppresses cadmium-induced neurotoxicity, in rats. The sole methanolic extract (56.24 ± 5.05 mg KAE/g; IC_50_: 1.90 mg /mL) was observed to exert antityrosinase activity.

All extracts displayed modest capacities to inhibit α-amylase, whereas the sole dichloromethane extract (2.28 ± 0.17 mmol ACAE/g; IC_50_: 0.80 mg/mL) showed moderate inhibitory activity against α-glucosidase. Srinivasulu et al. [46] demonstrated that the methanolic extract of *A. aspera* growing in India is able to inhibit α-amylase and enhance glucose uptake in the psoas muscle and adipose tissue of male Wistar rats. Protocatechuic acid is reported to show concentration-dependent inhibition of α-glucosidase and α-amylase [34]. Similarly, ferulic acid was effective in inhibiting α-amylase and α-glucosidase [39]. In another earlier in-vivo study, ferulic acid was able to suppress significantly blood glucose levels in streptozotocin-induced diabetic mice [47]. A general review about chlorogenic (5-caffeoylquinic) acid served to highlight the antidiabetic property of this compound [48]. Based on these findings, it can be noted that the inhibitory activities against all tested enzymes were dependent on the solvents used for extraction.

Herbals contain a wide range of bioactive molecules, many of which have biological activities. Aqueous and organic solvents (methanol, ethyl acetate, dichloromethane, etc.) are commonly used for extracting metabolites from plants. Nonetheless, due to the different polarities of molecules present in the plants, it is not always easy to identify the most suitable solvent for extracting the molecules of interest from the target plant. In fact, during extraction, solvents diffuse into plant material and solubilize the molecules having similar polarity. Accordingly, herbal drug preparations require the choice of a suitable solvent.

**Figure 3:**
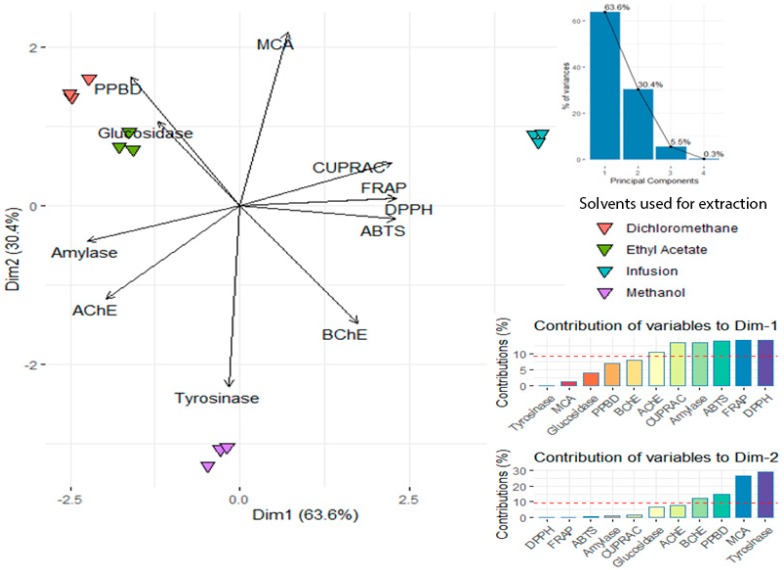
Unsupervised multivariate analysis with principal component analysis on *A. aspera* biological activities.

Thus, to assess the influence of solvent polarity on biological activities of *A. aspera*, a dataset was firstly submitted to the principal component analysis. Results depicted in Figure 3 elucidated that the two less polar solvents (dichloromethane and ethyl acetate), closed together, were distinguished from water and methanol along the first and second component of PCA respectively. The α-amylase inhibitory activity, both free radical scavenging and reducing power assays, characterizing PC1 (63.6% of total variance), allowed the separation of a water sample from dichloromethane and ethyl acetate extracts. However, tyrosinase, MCA, PPBD and BChE assays, associated with PC2 (30.4% of total variance), led to discriminate the methanol sample from dichloromethane and ethyl acetate samples. In light of the results, water extracts have the most significant antioxidant properties, suggesting that the polar compounds of *A. aspera* are responsible for such properties. Methanol was found to be more efficient in recovering specific compounds responsible for inhibition of tyrosinase, amylase and AChE enzymes, despite being inadequate for MCA activity. Besides, specific molecules involved in α-glucosidase inhibition and the total antioxidants were better extracted in DCM.

### 2.10. Prediction of Pharmacologic Targets and Pharmacokinetic Profile

**Figure 4:**
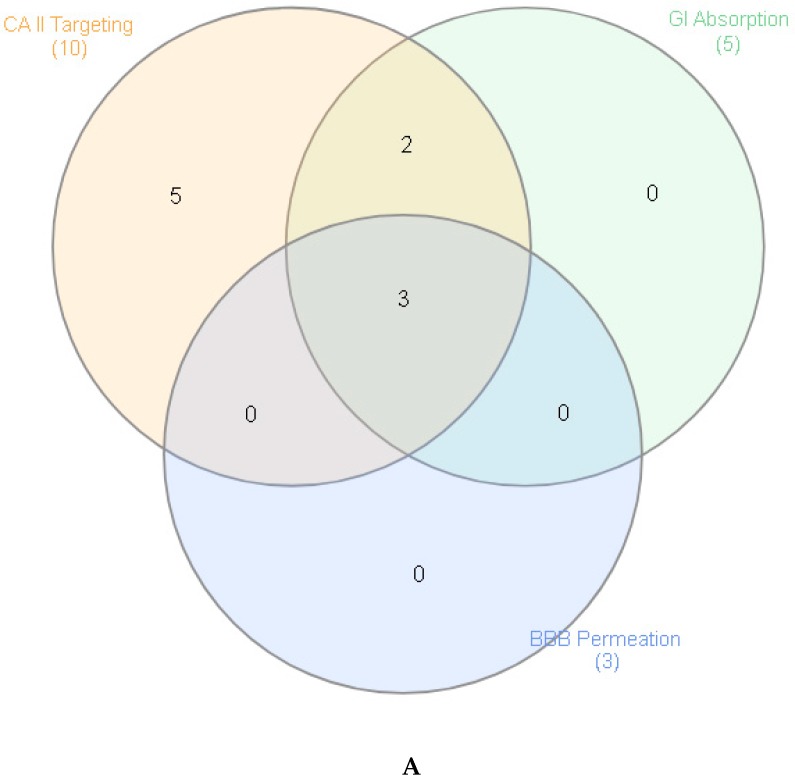

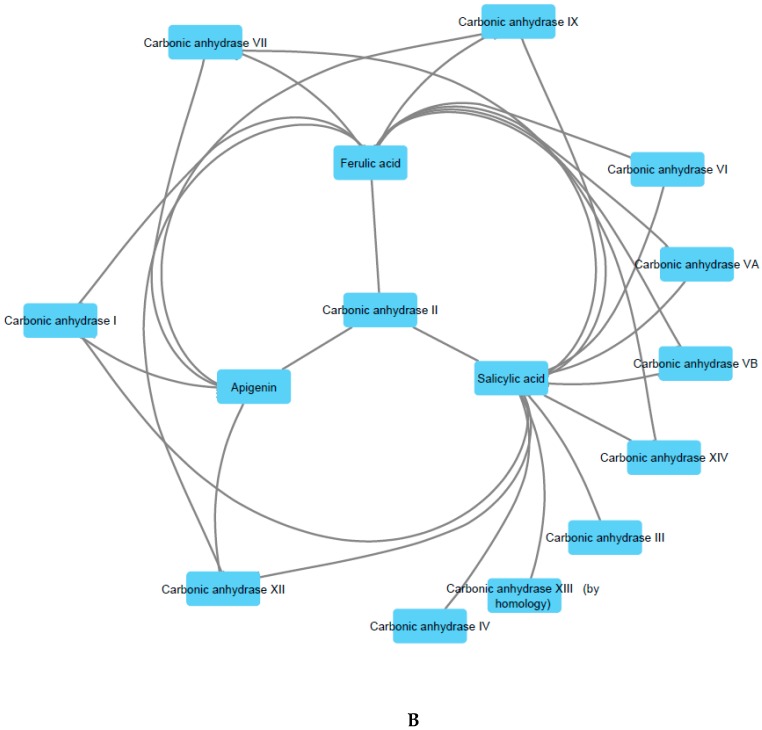
Pharmacological profile of phenolic compounds identified through ultra-high performance liquid chromatography coupled with hybrid quadrupole-Orbitrap high resolution mass spectrometry (UHPLC-HRMS) analysis in methanol, ethylacetate and dichloromethane extracts, and in water infusion of *A. aspera* dried aerial parts. Molecular target and pharmacokinetic profile were predicted through SwissTargetPrediction (http://www.swisstargetprediction.ch/) and SwissADME (http://www.swissadme.ch/index.php) platforms, respectively. Accordingly, the Venn diagram (**A**) was conducted based on high gastrointestinal absorption, blood barrier permeation and high probability to interact with a selected molecular target (carbonic anhydrase II). Finally, based on Venn diagram, components-targets analysis (**B**) was carried out through Cytoscape software (3.7.2 version) on apigenin, ferulic acid and salicylic acid. Extended results are included as Supplementary Materials (Supplementary Material Bioinformatic Analysis). **A**. Venn diagram related to selected phenolic compounds; **B**. Components-targets analysis. Single predicted carbonic anhydrase targets.

Considering the actual urge in searching alternative medications, including nutraceuticals and herbal preparations, which are characterized by a lower grade of side effects, it is of noteworthy interest to treat inflammatory conditions through extracts prepared from plants traditionally used by folk populations [49]. These extracts, especially those prepared with traditional and biocompatible solvents (water and hydroalcoholic solutions) in the forms of infusions or decoctions, could not only join efficacy and safety, due to their consolidated use in the population, but also represent innovative approaches for improving and valorizing local botanical resources and productive chains [50,51]. In this context, through a validated bioinformatic approach, we explored putative targets and pharmacokinetic profile of phenolic compounds, namely salicylic acid, caffeic acid, gentisic acid, kaempferol, apigenin, quinic acid, ferulic acid, chlorogenic acid, isoquercitrin and tiliroside, which were identified in extracts and infusion and that could be responsible for traditional uses of *A. aspera*, including diuretic, anti-inflammatory/antioxidant, antiproliferative, antiobesity and neuroprotective effects [4,11,15,16]. The chemical structure of each selected compound and the results of bioinformatic analysis are reported as Supplementary Materials (bioinformatic analysis folder), whereas the graphical representation of their putative pharmacological properties is depicted in Figure 4A,B. According to the bioinformatics screening, apigenin, ferulic acid and salicylic acid were highly absorbed. Additionally, the software’s prediction indicated their abilities to cross the blood brain barrier as well. This is consistent with literature [52,53], and could explain, albeit partially, the efficacy of *A. aspera* methanolic extract as a neuroprotective agent [16], whereas the antioxidant/anti-inflammatory role of apigenin, ferulic acid and salicylic acid could collectively contribute to explain the potential application of *A. aspera* extracts in treating chronic inflammatory diseases [54].

Interestingly, most of these compounds, namely apigenin, caffeic acid, ferulic acid, gentisic acid, isoquercitrin, kaempferol, salicylic acid and tiliroside showed moderate to high capacity to interact with several isoforms of carbonic anydrase (lyase family), among which it is noteworthy to highlight the type II isoform.

Salicylic acid was found to inhibit carbonic anhydrase II [55,56], and this could explain, albeit partially, not only its use as antiplatelet agent [57], but also the traditional use of *A. aspera* extracts in cardiovascular diseases [5,8,9]. Ferulic acid was also described as an inhibitor of carbonic anhydrase II, although its experimental IC_50_ value was at least 10-fold higher compared to acetazolamide, the reference enzyme inhibitor drug [58]. Despite there still being no specific studies, the putative interactions between carbonic anhydrase II and the other studied phenolic compounds, especially those characterized by a favorable pharmacokinetic profile, suggest further enzyme inhibition effects [59], that could explain, at least in part, the traditional use of *A. aspera* decoctions as diuretic agents, as well [4].

## 3. Materials and Methods

### 3.1. Plant Material and Preparation of Extracts

*A. aspera* was collected in a district of Yamoussoukro of Ivory Coast, Brobo city in the year 2019 and it was authenticated by the botanist Ouattara Katinan Etienne (Université Félix Houphouet Boigny, Abidjan, Ivory Coast). The aerial parts were divided and dried (in shade about 10 days). The dried samples were grounded by using a laboratory mill.

### 3.2. Extraction

In the present paper, we used different solvents including dichloromethane, ethyl acetate, methanol and water to obtain extracts. Maceration was used for dichloromethane, ethyl acetate and methanol. In this regard, plant materials (5 g) were macerated for 24 h at room temperature. Afterwards, mixtures were filtered and then solvents were removed by using rotary-evaporator. Regarding the water extract, plant material (5 g) was infused in the boiled water (100 mL). Then, water was removed by using freeze-drying. All extracts were stored at +4 °C until analysis. 

### 3.3. Chemical Profiling 

Mass analyses were carried out on a Q Exactive Plus mass spectrometer (Thermo Fisher Scientific, Inc., Waltham, MA, USA) equipped with a heated electrospray ionization (HESI-II) probe (Thermo Scientific, Waltham, MA, USA). Separation was achieved on a reversed phase column Kromasil EternityXT C18 (1.8 µm, 2.1 × 100 mm) column maintained at 40 °C. All chromatographic and analytical details are given in Supplementary Materials.

Colorimetric assays were conducted to determine total phenolic and flavonoid contents of *A. aspera* extracts and detailed methods were described in a previous paper of ours [60,61]. Total phenols and flavonoids were expressed as gallic acid (GAE) and rutin (RE) equivalents, respectively. 

### 3.4. Determination of Antioxidant and Enzyme Inhibitory Effects

The metal-chelating, phosphomolybdenum, ferric reducing power (FRAP), cupric reducing antioxidant capacity (CUPRAC), 2,2′-azino-bis (3-ethylbenzothiazoline-6-sulphonic acid) (ABTS) and 2,2-diphenyl-1-picrylhydrazyl (DPPH) activities of the extracts were evaluated following the methods described by Grochowski et al. [62]. Standard equivalents were used to evaluate antioxidant properties (trolox and EDTA). Enzyme inhibitory activities of the extracts against acetylcholinesterase (AChE), butyrylcholinesterase (BChE; by Ellman’s method), α-amylase, α-glucosidase and tyrosinase were assessed using standard in vitro bioassays, as previously described [57]. Enzyme inhibitory ability was evaluated as standard equivalents and IC_50_ values. 

### 3.5. Prediction of Putative Targets and Pharmacokinetics

Putative targets were identified through the bioinformatic method recently described by Gu and colleagues [63]. Briefly, chemical structures were prepared and converted in canonical SMILES using ChemSketch software. The SMILES were then processed by the Swiss Target Prediction (http://www.swisstargetprediction.ch/) and SwissADME (http://www.swissadme.ch/index.php) platforms, for predicting putative targets and pharmacokinetic profile, respectively. The name of identified targets was normalized according to the UniProt database (https://www.uniprot.org/). Finally, Cytoscape software (3.7.2 version, National Institute of General Medical Sciences (NIGMS), Bethesda, MD, USA) was used to create a Venn diagram of identified phytochemicals in the tested extracts and a component-target illustration network.

### 3.6. Statistical Evaluation

Results of replicate readings were pooled and presented as mean ± SD standard deviation. Firstly, principal component analysis (PCA) was done to evaluate the effect of extracting solvent on biological activities. One-way analysis of variance (ANOVA) and Tukey’s multiple range method were used to analyze the differences among samples. Differences at *p* < 0.05 were considered to be significant. R v 3.6.1 statistical software was used for the analysis.

## 4. Conclusions

Concluding, *A. aspera* extracts possess numerous secondary metabolites, including ferulic acid, apigenin and salicylic acid, with promising pharmacological applications in counteracting the burden of oxidative stress occurring in chronic inflammatory diseases, such as type 2 diabetes, cardiovascular and neurodegenerative disorders. On the basis of the present study, an improvement of the local chain production is desirable, also in view of more sustainable and circular economy. 

## Figures and Tables

**Table 1:** Peak assessment of metabolites in *Achyranthes aspera* extracts.

No	Tentative Structure		Molecular Formula	Exact Mass [M − H]^−^	Presence	Reference
		Fatty acids				
1	azelaic acid		C_9_H_16_O_4_	187.0968	1,2,4	[22]
2	undecanedioic acid		C_11_H_20_O_4_	215.1285	1,2,3	
3	dodecenedioic acid (traumatic acid)		C_12_H_20_O_4_	227.1289	1,2,3,4	
4	dodecanoic acid (lauric acid)		C_12_H_22_O_4_	229.1442	1,2,3,4	
5	linolenic acid		C_18_H_30_O_2_	277.2177	1	[23]
6	8-hydroxy-9,11,13-heptadecatrienoc acid		C_17_H_28_O_3_	279.1968	1,3	
7	12-hydroxy-6-heptadecenoic acid		C_17_H_30_O_3_	281.2123	1,3	
8	9-hydroxy-10,11,13,15-octadecatetranoic acid		C_18_H_28_O_3_	291.1970	1,3	
9	13-hydroxy-9,11,15-octadecatrienoic acid		C_18_H_30_O_3_	293.2119	1,2,3,4	
10	15-hydroxy-9,11,13-octadecatrienoic acid		C_18_H_30_O_3_	293.2126	1,2,3,4	
11	13-hydroxy-9,11-octadecadienoic acid		C_18_H_32_O_3_	295.2280	1,2,3,4	[23]
12	9-hydroxy-?-octadecenoic acid		C_18_H_34_O_3_	297.2438	1,3	[24]
13	15-hydroxy-9-oxo-10,12,14-octadecatrienoic acid		C_18_H_26_O_4_	305.1762	1,2,3,4	
14	14-hydroxy-9-oxo-11,13,15-octadecatrienoic acid		C_18_H_28_O_4_	307.1919	1,2,3,4	
15	9,10-dihydroxy-12,14,16-octadecatrienoic acid		C_18_H_30_O_4_	309.2076	1,2,3,4	
16	9,10-dihydroxy-11,13,16-octadecatrienoic acid		C_18_H_30_O_4_	309.2074	1,2,3	
17	9,10-dihydroxy-12,15,16-octadecatrienoic acid		C_18_H_30_O_4_	309.2074	1,2,3,4	
18	11,12-dihydroxy-9,14,15-octadecatrienoic acid		C_18_H_30_O_4_	309.2076	1,2,3,4	
19	15,16-dihydroxy-9,12-octadecadienoic acid		C_18_H_32_O_4_	311.2234	1,3,4	
20	9,10-dihydroxy-12,14-octadecadienoic acid		C_18_H_32_O_4_	311.2233	1,2,3,4	
21	9,10-dihydroxy-10,13-octadecadienoic acid		C_18_H_32_O_4_	311.2232	1,2,3,4	
22	12,13-dihydroxy-9-octadecenoic acid		C_18_H_34_O_4_	313.2389	1,2,3,4	[23]
23	9,10-dihydroxy-12-octadecenoic acid		C_18_H_34_O_4_	313.2388	1,2,3,4	[23]
24	9,10-dihydroxy-octadecanoic acid		C_18_H_36_O_4_	315.2544	1,2,3	[23]
25	9,10,13-trihydroxy-11,15-octadecadienoic acid		C_18_H_32_O_5_	327.2179	1,2,3,4	
26	9,12,13-trihydroxy-10,15-octadecadienoic acid		C_18_H_32_O_5_	327.2179	1,2,3,4	
27	11,12,15-trihydroxy-9,12-octadecadienoic acid		C_18_H_32_O_5_	327.2179	1,2,3,4	
28	11,12,13-trihydroxy-9,12-octadecadienoic acid		C_18_H_32_O_5_	327.2179	1,2,3,4	
29	9,10,15-trihydroxy-12,15-octadecadienoic acid		C_18_H_32_O_5_	327.2179	1,2,3,4	
30	11,12,15-trihydroxy-9,12-octadecadienoic acid		C_18_H_32_O_5_	327.2180	1,2,3,4	
31	9,10,13-trihydroxy,12-octadecenoic acid		C_18_H_34_O_5_	329.2338	1,2,3,4	[23]
32	9,12,13-trihydroxy,10-octadecenoic acid		C_18_H_34_O_5_	329.2338	1,2,3,4	[23]
		Carboxylic, phenolic and acylquinic acids, and phenylethanoid glycosides				
33	salicylic acid		C_7_H_6_O_3_	137.0230	1,2,3,4	
34	protocatechuic acid		C_7_H_6_O_4_	153.0184	1,2,3	*
35	gentisic acid		C_7_H_6_O_4_	153.0183	2,3,4	*
36	caffeic acid		C_9_H_8_O_4_	179.0338	1,2,3,4	*
37	quinic acid		C_7_H_12_O_6_	191.0552	1,2,3,4	
38	ferulic acid		C_10_H_10_O_4_	193.0498	1,2,3,4	*
39	salicylic acid-*O*-hexoside		C_13_H_16_O_8_	299.0776	1,2,3,4	
40	gentisic acid-*O*-hexoside		C_13_H_16_O_9_	315.0725	2,4	
41	chlorogenic (5-caffeoylquinic) acid		C_16_H_18_O_9_	353.0867	1,2,3,4	*
42	4-caffeoylquinic acid		C_16_H_18_O_9_	353.0872	3,4	
43	3,5-dicaffeoylquinic acid		C_25_H_24_O_12_	515.1218	1,2,3,4	*
44	4,5-dicaffeoylquinic acid		C_25_H_24_O_12_	515.1198	1,2,3,4	*
45	3,4,5-tricaffeoylquinic acid		C_34_H_30_O_15_	677.1532	1,2,3	*
		Flavonoids				
46	kaempferol-3-*O*-glucoside		C_21_H_20_O_11_	447.0941	1,2,3,4	*
47	isoquercitrin		C_21_H_20_O_12_	463.0891	1,2,3,4	*
48	apigenin-7-*O*-hexuronide-4’-*O*-rhamnoside		C_27_H_28_O_15_	591.1356	1,2,3,4	
49	kaempferol-3-*O*-neohesperidoside		C_27_H_30_O_15_	593.1522	1,2,3,4	
50	kaempferol-3-*O*-rutinoside		C_27_H_30_O_15_	593.1528	1,2,3,4	*
51	tiliroside		C_30_H_26_O_13_	593.1307	1,2,4	*
		Other compounds				
52	Roseoside corchoionoside C		C_19_H_30_O_8_	385.1884	1,2,3,4	
53	rubrosterone		C_19_H_26_O_5_	333.1680	1,2,3,4	
54	20-hydroxyecdysterone		C_27_H_44_O_7_	479.3018	1,2,3,4	[25]

*—compared to reference standard. 1—ethyl acetate, 2—methanol, 3—DCM and 4—infusion.

**Table 2:** Enzyme inhibitory properties of the tested extracts *.

		AChE Inhibition		BChE Inhibition		Tyrosinase Inhibition		α-Amylase Inhibition		α-Glucosidase Inhibition	
		mg GALAE/g	IC_50_ (mg /mL)	mg GALAE/g	IC_50_ (mg /mL)	Mg KAE/g	IC_50_ (mg /mL)	mmol ACAE/g	IC_50_ (mg /mL)	mmol ACAE/g	IC_50_ (mg /mL)
Extracts	DCM	3.97 ± 0.36 ^b^	0.68 ± 0.02 ^b^	na	na	na	na	0.66 ± 0.03 ^a^	1.69 ± 0.08 ^b^	2.28 ± 0.17	0.80 ± 0.06 ^a^
	EA	3.28 ± 0.21 ^c^	0.82 ± 0.05 ^c^	na	na	na	na	0.68 ± 0.01 ^a^	1.65 ± 0.01 ^b^	na	na
	Infusion	na	na	15.36 ± 0.22 ^a^	0.55 ± 0.01 ^b^	na	na	0.20 ± 0.01 ^b^	>5	na	na
	MeOH	4.87 ± 0.10 ^a^	0.55 ± 0.01 ^b^	16.00 ± 0.48 ^a^	0.53 ± 0.01 ^b^	56.24 ± 5.05	1.90 ± 0.17 ^b^	0.63 ± 0.04 ^a^	1.78 ± 0.12 ^b^	na	na
Standards	Galatamine		0.003 ± 0.0001 ^a^		0.008 ± 0.0001 ^a^		−		−		−
	Kojic acid		−		−		0.11 ± 0.01 ^a^		−		−
	Acarbose		−		−		−		0.72 ± 0.02 ^a^		1.04 ± 0.08 ^b^

* Values expressed are means ± S.D. of three parallel measurements. GALAE: Galatamine equivalent; KAE: Kojic acid equivalent; ACAE: Acarbose equivalent. Based on Tukey’s assay at *p* < 0.05, different letters (a, b, c and d) with same column indicated significant differences among the tested extracts (a: the highest ability). na: not active. − not tested.

## Supplementary Materials

The supplementary material and methods are available on online.
